# Lineage-specific elaboration of a conserved cnidogenic program drives cnidocyte diversification and illuminates cell type evolution

**DOI:** 10.21203/rs.3.rs-10009230/v1

**Published:** 2026-06-30

**Authors:** Benjamin Danladi, Layla Al-Shaer, Kejue Jia, Jamie A. Havrilak, Dylan Faltine-Gonzalez, Wyatt Forwood, Timothy DuBuc, Jacob Musser, Michael J Layden

**Affiliations:** 1 Department of Biological Science and Lehigh Oceans Research Center, Lehigh University, Bethlehem, PA, USA; 2 Department of Molecular and Cellular Biology, Yale University, New Haven, CT, USA; 3 Department of Biology, Queens College, The City University of New York, Flushing, NY, USA; 4 Biology and Biochemistry PhD Programs, The Graduate Center of the City University of New York, New York, NY, USA

## Abstract

Cnidocytes are a cnidarian synapomorphy that diversified considerably across the phylum, making them an excellent model to investigate how novel cell types emerge and evolve. By investigating the regulatory program patterning cnidocytes in the sea anemone *Nematostella vectensis*, we identified at least six molecular pathways contributing to the cnidogenic gene regulatory network, including a novel lineage-restricted transcription factor, *NvfoxE-like*, whose expression and targets are exclusive to cnidocytes. While *Nvznf845* and *NvpaxA* represent a conserved pan-cnidarian core program, *NvfoxE-like* and other lineage-specific genes represent elaborations restricted to hexacorallians or *Nematostella* specifically. Comparative analysis across cnidarian lineages reveals that only ~25% of cnidocyte-expressed genes are broadly conserved, while ~40% lack clear homologs outside *Nematostella*, consistent with substantial lineage-specific cnidogenic transcriptional profiles. While all cnidocytes have the same basic blueprint, there is extensive variation in harpoon morphology, venom composition, and capsule opening mechanisms. This suggests that rapid diversification of cnidocyte development occurred as new lineages arose. Our findings suggest that homologous cell types can share less than 25% transcriptional similarity, and perhaps less for more ancient cells, providing a molecular framework for understanding cell type diversification across evolution.

## Introduction:

Cnidocytes (stinging cells) are a defining synapomorphy of cnidarians (e.g., anemones, corals, jellyfish, hydra) that mediate prey capture and defense^1^. Their evolutionary novelty makes them a powerful model for understanding how new cell types emerge and diversify. Cnidocytes arise from progenitors that also generate neurons and neuro-glandular cells^2–4^. These progenitors express *SOX (SRY-related HMG-box)* family transcription factors, which are required for cnidocyte specification^4–6^. *Nvznf845* is the earliest known acting cnidocyte-specific regulator during cnidocyte differentiation in the sea anemone *Nematostella vectensis*, and it emerged at or near the base of the cnidarian clade^7^. In *Nematostella, Nvznf845* also regulates expression of another transcription factor *NvpaxA*, which is present in both *Hydra* and *Nematostella* cnidocytes^7–9^. These data suggest that the *sox → znf845 → paxA* pathway may represent a core conserved cnidogenic regulatory cascade in cnidarians.

After their emergence, cnidocytes underwent extensive diversification, giving rise to an impressive variety of subtypes and morphologies. All cnidocytes share a defining structural blueprint: a cell containing a pressurized, membrane-bound capsule (the cnidocyst) with an inverted tubule that can explosively evert outward upon mechanical or chemical stimulation^10^. In extant species, there are three main classes of cnidocytes: spirocytes (ensnaring), ptychocytes (adhesive), and nematocytes (piercing)^11^. Spirocytes are restricted to the anthozoans (e.g., corals and anemones) and have harpoons that lack barbed armature, mostly functioning to ensnare prey, and ptychocytes are specialized cnidocytes only found in the tube-building anemones, Ceriantheria^12–14^. Nematocytes are found widely across all cnidarian lineages, and are the most diverse group of cnidocytes^12,15^. For example, nematocytes can vary in harpoon morphology, venom composition, and capsule structures, creating incredible diversity within this class^16^. Certain morphological traits can also exhibit phylogenetic signatures, for example, the operculum, apical cap, and apical flaps, which are related to capsule opening, are restricted to specific lineages^17,18^. Taken together, the current observations argue that cnidocytes arose in the stem cnidarian and diversified into the variations observed in extant species, making them an exciting model to investigate the origin and evolution of novel cell types in animals.

Here, we utilized single-cell RNA sequencing (scRNA-seq) studies and functional approaches to identify targets of known and novel cnidocyte regulators that act early in cnidogenesis. By mapping these data to an existing single-cell atlas, we were able to elaborate on the initial cnidogenesis gene regulatory network (GRN) in the sea anemone *Nematostella vectensis*. Our findings confirmed the current model that the *znf845 → paxA* program is pan-cnidarian, which arose before or coincidentally with cnidocytes. Additionally, we identified a novel, lineage-specific, cnidocyte regulator, *NvfoxE-like* (formally called *NvfoxD3-like*)^20^. Investigation of the 989 target genes identified following the shRNA knockdown of *Nvznf845, NvpaxA*, and *NvfoxE-like* revealed six distinct regulatory programs/pathways that specify broad cnidocyte and subtype-specific characteristics in *Nematostella*. Further analysis of cnidarian orthogroup distributions for targets of each pathway found that only ~25% of the *Nematostella* cnidocyte genes are present across cnidarians, and ~40% of the genes are *Nematostella-specific*. These findings further elaborate the regulatory networks of cnidocyte specification in *Nematostella*, provide initial insights into how cnidogenic programs evolved, and set the stage for future studies to understand how evolutionary tinkering with the cnidogenic regulatory networks led to the diversification observed in extant species. Additionally, our findings argue for extensive diversification of cnidocyte development within cnidarian lineages and broadly imply that homologous cell types may have less than 25% similarity, which impacts our expectations of similarity when trying to homologize cell types across lineages.

## Materials and Methods:

### Animal care/ spawning:

All animals were maintained at 16°C in ⅓ strength artificial seawater with a salinity of 11–13ppt and a pH of 8.1 – 8.2 and given weekly water changes. To induce spawning, animals were transferred to a 25°C incubator overnight, with a light on from 11 pm onward, and moved to room temperature at 9 am. Fertilized embryos were de-gelled in 4% w/v cysteine solution^21^. Embryos used for experiments were cultured at 24°C and fixed at appropriate experimental timepoints in 4% paraformaldehyde following a previously published protocol^22^.

### shRNA-mediated gene knockdown:

RNAi-mediated gene regulation was utilized to knock down genes of interest. Primers for the shRNAs were designed as previously described (Additional file 1), and the shRNA transcription reaction was performed using an AmpliScribe T7-Flash Transcription Kit (Biosearch Technologies, Lucigen)^23^. We used a combination of two shRNAs to achieve knockdown for *NvfoxE-like*, and a single shRNA for *NvpaxA*, *Nvznf845*, *NvsoxC*, and *NvsoxB2*. An injection mix of 1 μg/μl shRNA, was microinjected into embryos immediately after fertilization and before the embryos started cleaving. For all gene knockdown experiments, control embryos were injected with equal concentrations of an established control shRNA^23,24^.

### Colorimetric *in situ* hybridization:

*In situ* hybridization was performed using a previously published protocol^25^. Probes were made by cloning a region of the mRNA of interest using gene-specific primers (Additional file 1), and a transcription reaction template was made from the cloned mRNA region. Digoxigenin RNA labelling mix (Roche 11277073910) was used for probe transcription reactions. Experiments were quantified by a researcher blind to treatment condition and were imaged with a Nikon DS-Ri2 color camera using the Nikon Element software and processed in Adobe Photoshop 2024.

### Transcriptomic analysis following gene knockdown:

Bulk mRNA sequencing was performed on gastrula-stage embryos (28hpf at 24°C) following RNAi-mediated gene knockdown as described above^23,26^. Each replicate consisted of ~1000 injected embryos. Five paired experimental and control replicates were sequenced for all knockdown experiments, except *for NvfoxE-like*, which had four replicates. RNA was stabilized by placing embryos from each replicate in 1 ml of TRI Reagent (Sigma-Aldrich) and then storing them at −20°C until total RNA was isolated, as previously described^27,28^. RNA concentration and purity were measured with an Agilent 2100 Bioanalyzer (all samples RIN ≥ 8.0). Library preparation, Illumina NovaSeq 6000 mRNA sequencing (paired end, 150bp reads), and read quality filtering and trimming were performed by Novogene Inc. as previously described^29^. Sequencing data can be retrieved from the National Center for Biotechnology Information’s Sequence Read Archive (GSE335078 - *NvfoxE-like* RNAseq, GSE335079 - *NvpaxA RNAseq*, and GSE335081 - *Nvznf845* RNAseq). The Galaxy web platform was used to map reads to the *Nematostella* Nvec200 genome and conduct differential gene expression analysis using DESeq2 as previously described^29–31^. A false discovery rate of *p* ≤ 0.05 and log2(FC) of ± 0.585 was used to determine differential gene expression for the *NvfoxE-like* and *NvpaxA* RNAseq, and an adjusted *p* ≤ 0.05 and log2(FC) of ± 0.585 was used for the *Nvznf845* RNASeq data analysis. Overlap between the targets identified in each RNAseq dataset was visualized using Biovenn^32^.

### Transgenesis and transgene analysis:

The *Nvncol3::mOrange* transgenic line was obtained from Yehu Moran’s lab^33,34^. The *NvfoxE-like*::*egfp* transgenic line was made using meganuclease-mediated transgenesis as previously described^33,34^. In short, we designed primers to clone the promoter region 2kb upstream of the *NvfoxE-like* transcription start site (Additional file 1). Z-stack images of F0 double transgenic embryos fixed 72 hours post fertilization (hpf) were taken using a Zeiss 880 laser scanning confocal microscope, and image processing was done using the Imaris 10.2 software.

### Single-cell RNA sequencing data analysis:

Downstream single-cell analysis was performed in R: A Language and Environment for Statistical Computing using the Seurat package (v5.1.0)^35^. This analysis utilized two pre-processed, publicly available, scRNA-seq datasets: an in-house developmental atlas spanning gastrula, early, mid, and late planula stages^36^, and a whole-organism atlas that includes adult tissues^37^. Global cell clusters across these datasets were visualized via UMAP projections using the DimPlot function.

Using our lab’s developmental dataset^36^, the DotPlot and FeaturePlot functions were used to screen for the spatial and relative expression of early-active transcription factors across cell populations, identifying specific regulators with enriched expression in cnidocytes (such as *NvfoxE-like*).

Following bulk RNA-seq analyses, we mapped our different gene groups—such as unique cnidogenic pathways, lineage-restricted genes, and broadly conserved groups—back to the single-cell landscapes. We used Seurat’s AverageExpression() function to calculate the average expression of these genes across the different single-cell clusters within both the global atlas (containing all cells) and the cnidocyte subset atlas. Finally, the pheatmap package was used to perform unsupervised hierarchical clustering to group genes based on their average expression across cell clusters and visualize them as a heatmap^38^.

### Phylogenetic analysis of Fox, Znf845, and PaxA:

We identified protein homologs of *N. vectensis* foxE-like, znf845, and paxA using a combination of BLASTp (version 2.15.0)^39^ and PROST^40^, a protein language model-based approach for remote homology detection. We performed searches against six cnidarian species (*Acropora digitifera*, *Acropora tenuis*, *Hydra vulgaris*, *Nematostella vectensis*, *Stylophora pistillata*, and *Xenia sp*.) and four model organisms (*Homo sapiens*, *Mus musculus*, *Drosophila melanogaster*, and *Caenorhabditis elegans*). BLASTp searches were performed using the BLOSUM62^41^ amino acid substitution matrix, with default gap penalty parameters (11 for gap opening and 1 for gap extension). Both BLAST and PROST results were filtered using an e-value cutoff of 1e-6. Multiple sequence alignments were generated using MAFFT (version v7.471) with a maximum iteration setting of 1000 to refine the alignment and using a local pairwise focus to emphasize the conserved regions of the sequences. Phylogenetic trees were then generated with IQ-TREE (version 2.3.6) with default parameters and 1000 bootstrap replicates.

### Identification of Fox target ortholog expression:

We identified orthologs of target genes for the *foxE-like* transcription factor across cnidarians with OrthoFinder (version 3.0.1b1) using default parameters^42^. Expression patterns were assessed in previously published datasets for the anthozoans *Nematostella vectensis*, *Stylophora pistillata*, and *Xenia sp*., and the medusozoan *Hydra vulgaris*^43,44^. For *Stylophora*, *Xenia*, and *Hydra*, we used dataset versions curated by Levy et al. (2021)^44^, which generated metacell and cell type family assignments, which were used for plotting. For each target gene ortholog, we generated bar plots and violin plots of raw counts in Seurat (version 5.1.0) to illustrate the cell type and family-specific expression profiles^45^.

### Phylogenetic analysis of cnidarian Fox genes:

Forkhead (Fox) gene sequences were identified from publicly available genomic and transcriptomic resources. Cnidarian gene models were obtained from either the NCBI Genomes resource or Ensembl^46,47^. Our dataset included 15 cnidarian species spanning anthozoans, scyphozoans, cubozoans, hydrozoans, and staurozoans. To root the phylogeny and provide a broader metazoan context, we included outgroup species from sponges, ctenophores, placozoans, flatworms, nematodes, arthropods, chordates, and one mesozoan species. Outgroup taxa included *Amphimedon queenslandica*, *Spongilla lacustris* (Porifera), *Mnemiopsis leidyi* (Ctenophora), *Trichoplax* sp. H1 (Placozoa), *Schmidtea mediterranea* (Platyhelminthes), *Caenorhabditis elegans* (Nematoda), *Drosophila melanogaster* (Arthropoda), *Mus musculus* and *Homo sapiens* (Chordata), and *Rhopalura muelleri* (Mesozoa: Orthonectida).

Candidate Fox genes were identified using BLASTp and tBLASTn searches against genome and transcriptome assemblies using known forkhead protein sequences as queries. For species lacking well-annotated gene models, putative gene regions were predicted using AUGUSTUS and manually curated when necessary^48^. The presence of forkhead domains was confirmed using the SMART protein domain prediction tool^49^.

Full-length protein sequences were aligned using MUSCLE within MEGA11, using default parameters^50,51^. The alignment was visually inspected and manually curated to correct potential misalignments, particularly in highly variable or gapped regions. No trimming or domain-specific filtering was applied; the entire protein sequences were retained for phylogenetic analysis.

Phylogenetic reconstruction was conducted using MrBayes v3.2.7, on the CIPRES Science Gateway^52,53^. Bayesian inference was performed using the WAG amino acid substitution model with a gamma-distributed rate variation across sites. Two independent runs, each with four Markov chains, were executed for 1,000,000 generations, with sampling every 100 generations. The first 25% of samples were discarded as burn-in. Convergence was assessed by confirming that the average standard deviation of split frequencies was below 0.01. The resulting consensus tree was visualized using FigTree v1.4.4 (http://tree.bio.ed.ac.uk/software/figtree/). Posterior probability values were annotated on internal nodes to indicate clade support. The final tree was used to assess orthologous and paralogous relationships of Fox genes across cnidarian lineages and other metazoan taxa.

### Reciprocal best hit search:

We used the blast_best_reciprocal_hit function of the metablast function on R to identify reciprocal best hits between *Nematostella vectensis*, hexacorals *Exaiptasia diaphana* and *Stylophora pistillata*, the octocoral *Xenia sp*., and the hydrozoan *Hydra vulgaris*^54^. The blastp task was used, and an expectation value (e) threshold of 1e^−6^ was used as a cut-off selection of putative orthologs. *Nematostella* protein sequences were accessed from the genome assembly hosted by the Stowers Institute of Medical Research^31^, and sequences for *H. vulgaris* strain AEP were accessed from the genome assembly hosted by the National Human Genome Research Institute^55^. For comparisons to *octocorallia*, we used gene models hosted on NCBI for *Xenia sp*. (RefSeq assembly: GCF_021976095.1)^56^. For comparisons to *hexacorallia*, we used gene models hosted on NCBI for *Aiptasia CC7* and *Stylophora*^57,58^.

## Results:

### *NvfoxE-like* is a novel promoter of cnidocyte fates.

**Figure 1.**
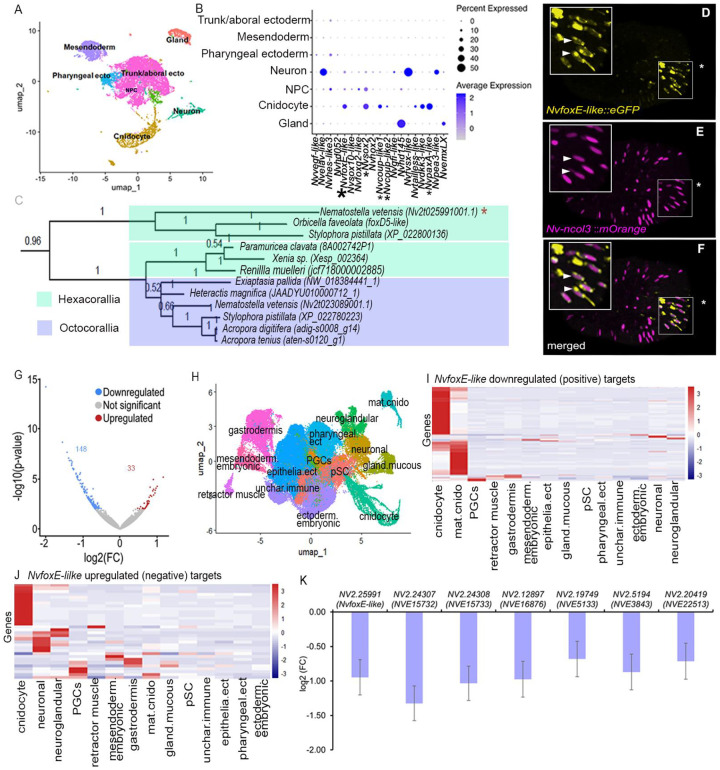
The transcription factor *NvfoxE-like* is an important regulator of cnidogenesis. Single-cell atlas showing cnidocyte cell states in *Nematostella vectensis* at gastrula stage(A). Dot-plot shows transcription factor expression in different cell types (B), small asterisks show transcription factors with known cnidocyte expression and the large asterisk show *NvfoxE-like*. Phylogenetic analysis of *NvfoxE-like* with other proteins containing the forkhead DNA binding domain across Cnidaria shows *NvfoxE-like* is restricted the hexacoral lineage (C), red asterisk shows position of *NvfoxE-like*. A double transgenic line of *NvfoxE-like*::eGFP and *Nvncol3*::mOrange showing colocalization (white triangles) in a planula stage embryo (D-F). The white asterisk in panels D-F marks the oral end of the embryo. Volcano plot showing distribution of genes that change expression after knockdown of *NvfoxE-like* (G). Single-cell sequencing atlas of *Nematostella vectensis* showing all cell-types in at different embryonic stages and in adults (H). Heatmap showing genes with reduced expression after knockdown of *NvfoxE-like* are mainly enriched in cnidocytes (I), and genes with increased expression are enriched in cnidocytes as well as neuronal and germ cells (J). Six out of ten previous identified cnidocyte enriched genes have reduce expression after knockdown of *NvfoxE-like* (K). Genes were considered differentially expressed if the log2(FC) change was ± 0.585 and *p* ≤ 0.05.

To identify putative novel cnidocyte regulators in *Nematostella*, a list of early-expressed transcription factors was generated by screening for their expression in cnidocytes using our *Nematostella* scRNA-Seq developmental atlas (Figure 1A – B)^20,36^. In addition to known cnidocyte genes (*NvpaxA, Nvsox2, Nvcoup-like1* (aka *NR12*^*7*^)*, Nvdkk3-like3*), *NvfoxD3-like* (aka *NvfoxE-like*) was also found to be highly enriched in cnidocytes (Figure 1B, large asterisk)^20,5,7,19,59^. Phylogenetic analyses of *NvfoxD3-like* with known cnidarian and bilaterian *fox genes* revealed that it strongly groups with the foxE gene family rather than the foxD family, which appears to have undergone several gene duplications in early cnidarian evolution (Figure S1A–B, Additional file 2). Hence, we refer to *NvfoxD3-like* as *NvfoxE-like* onwards. To better describe the distribution of *NvfoxE-like* within cnidarians, we performed further phylogenetic analysis of forkhead domain–containing genes across Cnidaria (Figure 1C, Additional file 3). The *NvfoxE-like* gene arose as a duplication only within the hexacorals (Figure 1C).

*NvfoxE-like* expression is restricted to cnidocytes and their progenitors in single-cell atlases generated throughout development (Figure 1B, Figure S1C – H)^36^. mRNA *in situ* hybridization (ISH) confirmed a scattered salt-and-pepper pattern throughout development in the body column and tentacles when cnidocytes are forming (Figure S1I–K)^53^. Similarly, we injected the *NvfoxE-like::egfp* transgene into embryos from the *Nvncol3::morange* broad cnidocyte reporter line and found significant overlap between the mosaic expression of the F0 *NvfoxE-like* reporter and *Nvncol3* expressing cells (Figure 1G–I)^34^. All observations are consistent with *NvfoxE-like* being expressed in developing cnidocytes and their progenitors.

To determine if *NvfoxE-like* regulates cnidocyte development, bulk RNAseq was conducted on gastrula-stage embryos injected with shRNA targeting *NvfoxE-like* or a scrambled control shRNA (n=4 replicates each, 1000 embryos per replicate)^23,24^, and differentially expressed genes (DEGs) were identified between the two samples. There were 148 positive target genes (downregulated) and 33 negative target genes (upregulated) in the *NvfoxE-like* shRNA-injected animals (Figure 1G, additional file 4). A subset of genes was chosen for ISH and/or qPCR validation from the positive target list by ranking DEGs based on their adjusted p-value significance then dividing them into terciles. We successfully generated *in situ* hybridization probes and/or qPCR primers that covered 13 genes from our list. These genes represented seven genes from the first tercile, two from the middle, and four from the third. shRNA injections were repeated in new batches of animals that were either fixed for *in situ* or used for RNA isolation for qPCR. All six genes tested by *in situ* hybridization exhibited salt-and-pepper expression in control animals, which decreased following *NvfoxE-like* knockdown (Figure S2 A–G). Similarly, all but one gene assessed by qPCR following *NvfoxE-like* knockdown showed decreased expression relative to the control (Figure S2H). All 181 *NvfoxE-like* targets were then mapped to a *Nematostella* single-cell atlas^37^, revealing that the majority of the *NvfoxE-like* targets are enriched or exclusively expressed in cnidocytes (Figure 1 H–J), with some neuronal and germ cell expression for the negative targets only^60^. It is worth noting that a recent study identified ten genes whose expression was highly enriched in *Nematostella* cnidocytes^34^; of those ten genes, six are positive targets of *NvfoxE-like* (Figure 1 K). These data indicate that *NvfoxE-like* is required for the expression of cnidocyte-specific genes.

### *NvfoxE-like* functions in parallel to the *znf845→paxA* program.

**Figure 2:**
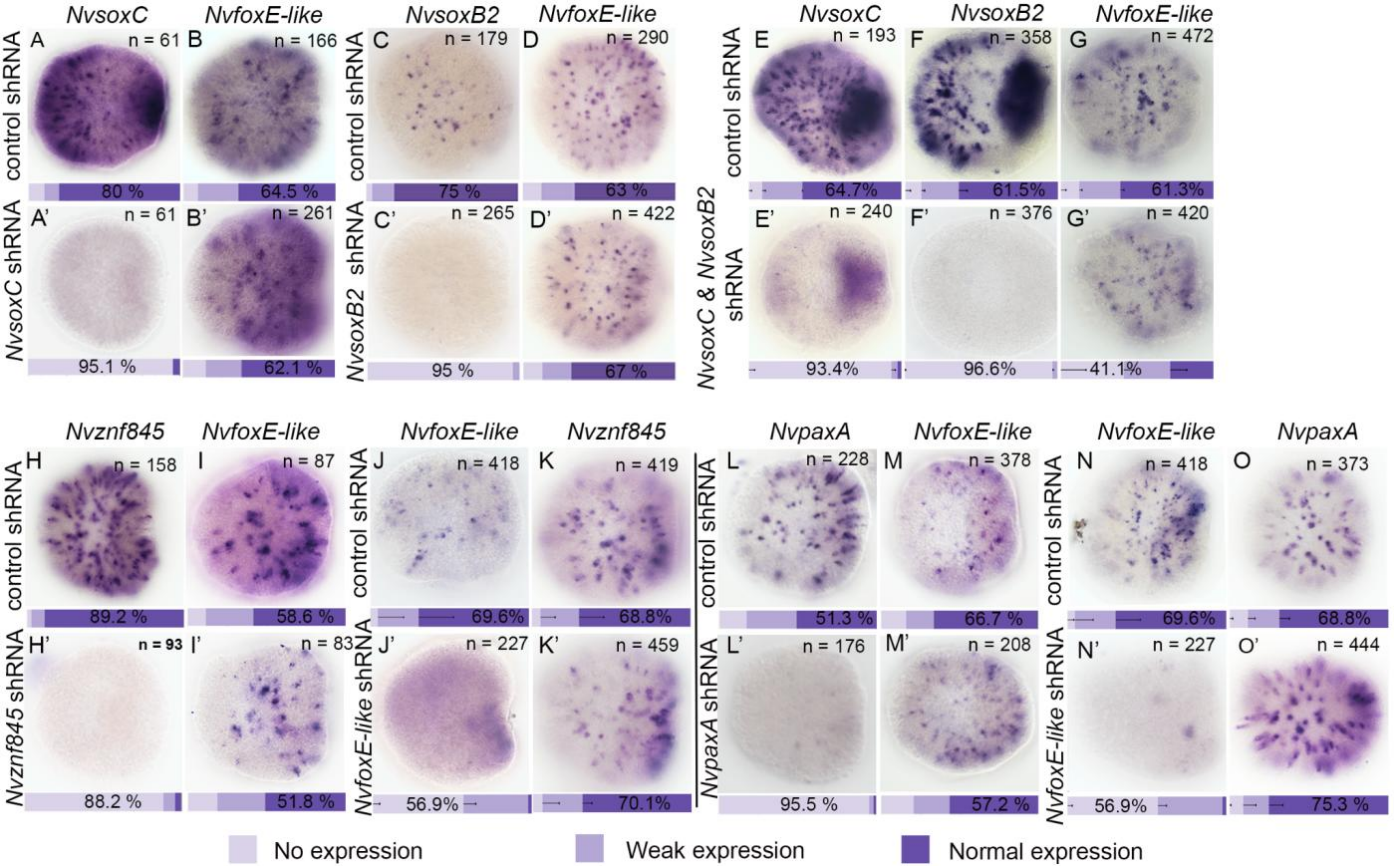
*NvfoxE-like* does not regulate cnidogenesis downstream of *NvsoxC* and *NvsoxB2*. mRNA expression xpression levels of *NvfoxE-like* did not change after shRNA mediated knockdown of *NvsoxC* (A - B), and *NvsoxB2* (C - D). Expression of *NvfoxE-like* is only slightly reduced after double knockdown of *NvsoxC* and *NvsoxB2* (E - G). shRNA mediated knockdown of *Nvznf845* did not affect expression of *NvfoxE-like* (H - I), similarly, knockdown of *NvfoxE-like* did not affect expression levels of *Nvznf845* (J - K). mRNA expression level of *NvfoxE-like* did not change after shRNA mediated knockdown of *NvpaxA* (L - M), and knockdown of *NvfoxE-like* slightly increased expression of *NvpaxA* (N - O). Quantification in the bar represent percentages with the most prevalent phenotype labled with the numerical value. Error bars represent standard error from three technical replicates for *NvsoxC* and *NvsoxB2* knockdown experiments, and error bars represents standard error from two technical replicates for *Nvznf845* and *NvpaxA* knockdown experiments.

To determine if *NvfoxE-like* functioned within the known cnidogenesis pathway, several epistasis experiments were performed. First, expression of *NvfoxE-like* was assessed following knockdown of the *sox* genes (*NvsoxC, NvsoxB2*), *Nvznf845*, and *NvpaxA*. Surprisingly, reducing *NvsoxC, NvsoxB2*, or both reduced *NvfoxE-like* expression, but the effect was much less significant than what is reported for *Nvznf845* or *NvpaxA*, which are effectively absent following knockdown of either *sox* gene (Figure 2 A – G, Figure S2 I–J)^5,7,59^. This implies that there are additional inputs to promote *NvfoxE-like* expression.

To determine whether *NvfoxE-like* interacts with the *Nvznf845* and *NvpaxA* cnidogenic transcription factors, the expression of each gene was assessed across different knockdown backgrounds (Figure 2 H–O). Knockdown of *Nvznf845* did not affect *NvfoxE-like* expression (Figure 2 H – I), and *Nvznf845* expression was not disrupted following *NvfoxE-like* knockdown (Figure 2 J – K). Similarly, the loss of *NvpaxA* minimally disrupted expression of *NvfoxE-like* and *vice versa* (Figure 2 L–O, Figure S2K). These findings were recapitulated with our RNAseq results for these three genes, indicating no interaction between *NvfoxE-like* and *NvpaxA* (See below and Additional files 2–3). Collectively, these data argue that *NvfoxE-like* functions in parallel to, or redundantly with, the known *znf845→paxA* cnidogenic program, and that *NvfoxE-like* may require inputs beyond the *sox* transcription factors known to be expressed in cnidocyte progenitors^4,5,61^.

### *Znf845* and *paxA* are conserved cnidocyte-specific genes.

*Nvznf845* and *NvpaxA* homologs are both required for cnidocyte development in *Nematostella*, and *znf845* homologs are currently found only in cnidarians^7^. To assess if these represent cnidocyte-specific genes, and likely function in cnidogenesis throughout the cnidarians, their expression was quantified in existing scRNA-seq databases for species that represent the hexacoralian and octocoralian anthozoans, as well as one medusozoan (Figure S3)^43,44,56^. Both genes are expressed exclusively in cnidocytes in both *Nematostella* and the coral *Xenia sp*. (Figure S3 A–D). In *Hydra, znf845* is expressed exclusively in cnidocytes and precursor cells (Figure S3E), but *paxA* is expressed in cnidocytes and an uncharacterized cell type (Figure S3F). Collectively, these data support the previous hypothesis that *znf845*, and likely represents a core component of the ancestral cnidocyte regulatory program^7^. These data support the existing model that these genes have a pan-cnidarian role in cnidogenesis, and that they likely represent a core kernel of the conserved pan-cnidarian cnidogenic GRN.

### Multiple cascades promote Nematostella cnidogenesis.

**Figure 3:**
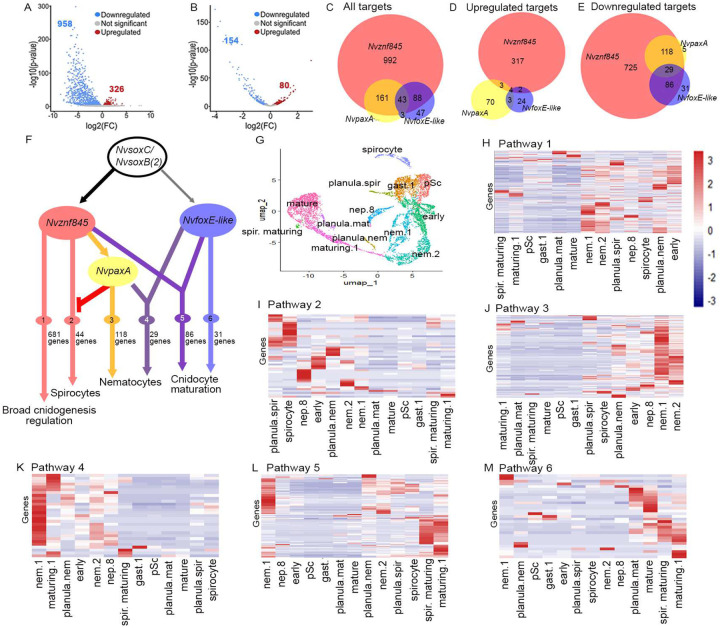
Multiple gene regulatory pathways contribute to the cnidogenic gene regulatory program. Volcano plots showing genes differentially expressed after knockdown of *Nvznf845* (A) and *NvpaxA* (B). Venn diagrams showing the overlap between bulk RNA-sequencing targets of *NvfoxE-like*, *NvpaxA*, and *Nvznf845* for all differentially expressed genes (C), downregulated targets (genes with reduced expression after shRNA-mediated knockdown) (D), and upregulated targets (genes with increased expression after shRNA-mediated knockdown) (E). Proposed gene regulatory network (GRN) of cnidogenesis, illustrating at least six regulatory pathways contributing to the GRN (F). Single-cell sequencing atlas of *Nematostella vectensis* showing all cnidocyte subtypes and cell states (G). Heatmap showing genes uniquely regulated by *Nvznf845* are enriched in most cell types (Arrow 1 in F), including nematocytes and spirocytes, but reduced in maturing and matured cnidocytes (H). Genes whose expression is promoted by *Nvznf845* but repressed by *NvpaxA* (Arrow 2 in F) are enriched in spirocytes, Nep8+ nematocytes, and early cnidocytes (I). Genes co-regulated by *Nvznf845* and *NvpaxA* (Arrow 3 in F) are enriched mainly in nematocytes (Nem1, Nem2, and planula nematocytes) (J). Genes co-regulated by *Nvznf845*, *NvpaxA*, and *NvfoxE-like* (Arrow 4 in F) are enriched mainly in maturing and Nem1 cnidocytes (K). Genes co-regulated by *Nvznf845* and *NvfoxE-like* (Arrow 5 in F) are enriched in maturing cnidocytes and Nem1 cells (L). Genes uniquely regulated by *NvfoxE-like* (Arrow 6 in F) are enriched in maturing and matured cnidocytes (M).

Because the *Nvznf845 → NvpaxA* pathway and *NvfoxE-like* genes do not interact, we sought to identify whether they have unique or shared targets. Targets of *Nvznf845* and *NvpaxA* were identified using the same shRNA-mediated gene knockdown and bulk RNAseq approach used for *NvfoxE-like* explained above. We found 958 positive and 326 negative targets of *Nvznf845* (Figure 3 A), and 154 positive and 80 negative targets of *NvpaxA* (Figure 3 B, Additional file 4). Similarly, we mapped the positive and negative targets to a *Nematostella* single-cell atlas (Figure S3G–K)^37^. Not surprisingly, nearly all of the positive targets are highly enriched in, or specific to, cnidocytes for both *Nvznf845* and *NvpaxA* (Figure S3H–I). The negative targets of *Nvznf845* are overrepresented in mature cnidocytes, uncharacterized immune cells, neuronal cells, and neuro-glandular cells (Figure S3J). The negative targets of *NvpaxA* are mainly restricted to cnidocytes (Figure S3K).

Shared and unique targets between *Nvznf845*, *NvpaxA*, and *NvfoxE-like* were identified by comparing the overlap of all target genes (Figure 3 C), the overlap of only the up-regulated genes (Figure 3 D), and the overlap of only the down-regulated genes (Figure 3 E). These three transcription factors function in at least six regulatory cascades, which are described below (Figure 3 F, Additional file 5). We focused on cascades containing at least 10 genes. Most shared targets were regulated the same way by all the upstream inputs. The exceptions were 44 genes that are suppressed by *NvpaxA* but require *Nvznf845*. Overall, the shared and unique target comparisons suggest that *Nvznf845* is required for five of the six regulatory cascades that promote cnidocyte development. First, 681 of the 958 genes that require *Nvznf845* for expression are unique to *Nvznf845* (Figure 3 F, salmon-pink arrow). The previously identified *Nvznf845 → NvpaxA* pathway is required for the expression of 147 genes and is described by two pathways. One which requires *Nvznf845* and *NvpaxA* (118 genes; Figure 3 F, orange arrow), and a second that also receives inputs from *NvfoxE-like* (29 genes; Figure 3 F, light purple arrow). A fourth cascade requires input from *NvfoxE-like* and *Nvznf845*, for the expression of 86 genes (Figure 3 F, purple arrow), and the fifth *Nvznf845*-dependent pathway includes 44 genes that receive positive input from *Nvznf845*, and a negative input from *NvpaxA* (Figure 3 F, salmon-pink arrow 2). Additionally, previously published cnidocyte genes were cross-referenced against the target lists for these transcription factors (Table S1). While most of the genes (32/42) could be assigned to one of our cascades, 10/42 genes were not targets of any of the transcription factors we described here, implying that additional cnidogenic inputs remain undiscovered.

To determine what role each of the six pathways plays in cnidogenesis, the genes in each were mapped onto a cnidocyte single-cell atlas (Figure 3 G–M)^37^. The genes that only require *Nvznf845* (indicated by the salmon-pink arrow in Figure 2 F) are broadly distributed across multiple cnidocyte cell populations but are noticeably not expressed in maturing cnidocytes (Figure 3 H). This broad distribution of target expression is consistent with previous observations that *Nvznf845* plays a critical role early in cnidogenesis^7^. Genes requiring positive *Nvznf845* input but are repressed by *NvpaxA* (indicated by the second salmon-pink arrow in Figure 3 F) are enriched in spirocytes and Nep 8+ cnidocytes (Figure 3 I). The enrichment of genes from this cascade in the spirocytes aligns with existing literature, which posits that *NvpaxA* functions downstream of *Nvsox2*, a pathway known to repress the acquisition of spirocyte-like phenotypes^19^. Genes in the cascade that require both *Nvznf845* and *NvpaxA* (indicated by the orange arrow in Figure 3 F) have restricted expression within the nematocyte cell populations (Figure 3 J). Genes that require *NvfoxE-like*, *Nvznf845*, and *NvpaxA* are also enriched in nematocytes as well as maturing cnidocytes (cascade indicated by the light purple arrow in Figure 3 F, Figure 3 K). Finally, the cascades requiring both *Nvznf845* and *NvfoxE-like* (indicated by the purple arrow, Figure 3 F), or only *NvfoxE-like* input (indicated by the blue arrow in Figure 3 F), both exhibit enriched expression profiles in mature and maturing cnidocytes (Figure 3 L–M). These findings suggest that these three transcription factors represent at least 6 regulatory cascades that specify cnidocytes in *Nematostella*, and each cascade can be assigned functions in generic cnidocyte phenotypes, cnidocyte subtype identity, or cnidocyte maturation.

### Lineage-specific expansion of cnidogenic genes supplements a broadly conserved cnidogenic program across Cnidaria.

**Figure 4:**
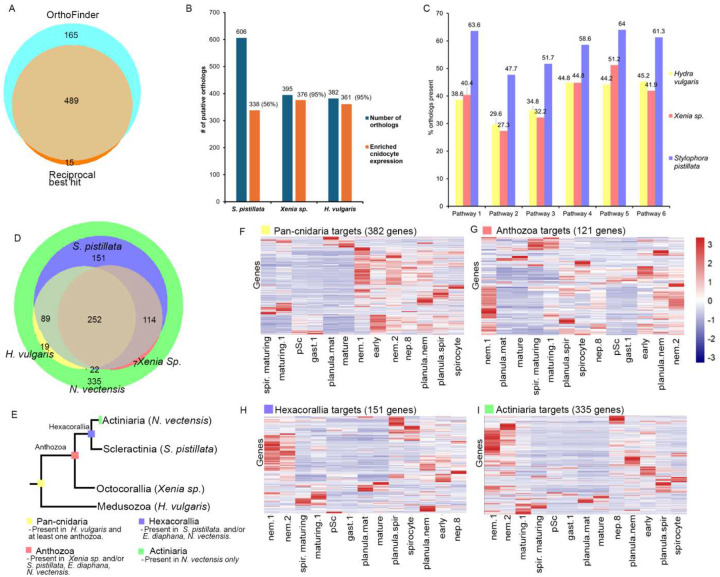
Lineage-specific expansion of cnidocyte transcriptome contribute to cnidocyte diversity across cnidaria. Venn diagram showing the overlap between orthologs identified by OrthoFinder and reciprocal best hit (RBH) approaches (A). Bargraph showing number of orthologs identified in each species, with the number of orthologs with enriched expression in cnidocytes cells using scRNA-seq atlas od same specie (B). Bar graphs showing the distribution of orthologs identified by OrthoFinder across six cnidogenic pathways in *H. vulgaris*, *Xenia* sp., and *S. pistillata* (C). Venn diagram showing the overlap between *N. vectensis* cnidogenic GRN orthologs identified in *H. vulgaris*, *Xenia* sp., and *S. pistillata* (D). Cnidarian phylogeny showing distribution of target gene orthologs classified by phylogenetic depth: pan-cnidarian, anthozoan-specific, hexacoral-specific, or Nematostella vectensis-specific (E). Heatmaps showing average expression of cnidocyte GRN target genes grouped by phylogenetic conservation across cnidarian lineages: pan-cnidarian (F), anthozoan (G), hexacorallian (H), and Nematostella-specific (I) orthologs.

Given that *Nvznf845* and *NvpaxA* are conserved cnidocyte genes across cnidarians, and *NvfoxE-like* is only found within hexacorallians, we sought to determine how their targets are conserved in different lineages. Homologs for the target genes in each pathway/cascade were identified using both reciprocal BLAST and OrthoFinder in phylogenetically informative species that possessed scRNA-seq atlases (Figure 4, additional files 5 and 6). *Hydra vulgaris* represented medusozoans, octocorals were represented by *Xenia sp*., and within the hexacorals, *Stylophora pistillata* represented the scleractinians and *Nematostella vectensis* represented the actiniarians^62,63^.

OrthoFinder recovered more shared orthogroups across species than reciprocal BLAST identified homologs, with nearly 100% overlap between the two methods (Figure 4 A, Figure S4A). Assessing the orthogroups identified by OrthoFinder, the most closely related species, *Stylophora pistillata*, shared the most orthogroups with *Nematostella* (606/989 – 61.3%; Figure 5B), whereas fewer were found in the more distant octocoral and medusozoan relatives (40% in *Xenia* and 38.6% in *Hydra*; Figure 4B). This gradient in shared orthogroups corresponds to phylogenetic distance from *Nematostella*, with hexacorallians recovering more than octocorals or medusozoans. Of the homologs identified, 95% were enriched in cnidocytes when assessed against existing scRNA-seq atlases for each representative species, suggesting that the identified homologs represent conserved cnidocyte genes across Cnidaria rather than broadly expressed genes (Figure 4 B).

One surprising observation was that for the 989 *Nematostella* genes assessed (the combined non-redundant targets of *NvfoxE-like*, *Nvznf845*, and *NvpaxA* enriched in cnidocytes), 383 (38.7%) had no detectable homologs in the species examined, suggesting these may represent lineage-specific genes, though highly diverged homologs cannot be excluded without further analysis. To resolve whether these genes are *Nematostella*-specific or actiniarian-specific, we performed a reciprocal BLAST of the target genes with another actiniarian species, *Exaiptasia diaphana*. The gene profile recovered for *Exaiptasia* was more similar to *Nematostella* than to other cnidarians, and we observed a slight increase in the number of homologs identified compared to other hexacorallians (Figure S4A–B), but approximately 40% of genes still lacked detectable homologs, suggesting these represent *Nematostella*-specific genes.

Homologs were found for genes in all six of our identified pathways across all cnidarian species considered, consistent with the presence of all six cascades in the stem cnidarian (Figure 4 C, Figure S4A). This finding was notable given the absence of *NvfoxE-like* outside of hexacorallians — one of the transcription factors identified as regulating several of these cascades in *Nematostella*. The pan-cnidarian conservation of cascade components despite the hexacoral-restricted distribution of *NvfoxE-like* suggests that the transcriptional regulation of these cascades may differ across cnidarian lineages even where the downstream effector genes are conserved, though the identity of potential ancestral regulators in non-hexacorallian lineages remains to be determined.

To identify whether genes conserved at different phylogenetic depths — pan-cnidarian, anthozoan, hexacorallian, or actiniarian (Figure 4 D–E) — preferentially mark specific cnidocyte subtypes, the genes conserved from each node were mapped to the *Nematostella* scRNA-seq atlas^37^. Mapping each gene set onto the cnidocyte subset of the single-cell atlas did not reveal a cnidocyte subtype pattern associated with conservation at any specific phylogenetic depth (Figure 4 F–I; Figure S4D–H). This was true both at the level of all conserved genes and when each of the six cascades was considered independently (Figure S5A–H). There was however, a consistent decrease in enrichment of conserved genes across all phylogenetic depths in proliferative progenitors, including gast1 and pSC cell populations, and in maturing and mature cnidocyte populations (Figure 4 F–I, Figure S4D–H), suggesting that the most phylogenetically conserved components of the cnidogenic program are preferentially expressed in intermediate rather than terminal differentiation states. The absence of a subtype-specific enrichment pattern across phylogenetic depths may reflect the limitations of morphologically defined cnidocyte subtype classifications for capturing functionally relevant gene expression differences, a possibility we address in the *Discussion*.

Collectively, these findings demonstrate that the transcriptome of *Nematostella vectensis* contains both a broadly conserved core, present across cnidarian lineages and enriched in cnidocytes, and a substantial lineage-specific component that is expanded in *Nematostella* relative to other species examined. This pattern is consistent with a model in which lineage-specific expansions of the cnidocyte transcriptome accompanied the diversification of cnidarian lineages^64^, contributing to the diversity of cnidocyte phenotypes observed across Cnidaria.

## Conclusion:

Investigating the regulatory targets of *NvfoxE-like*, *Nvznf845*, and *NvpaxA* in *Nematostella vectensis* identified at least six regulatory cascades specifying both generic cnidocyte features and subtype-specific characteristics, though the absence of several known cnidocyte-enriched genes from the target list implies that additional early inputs into cnidocyte specification remain to be identified (Table S1). Among the three regulators, *NvfoxE-like* is a lineage-restricted regulator that arose through gene duplication within hexacorals, with its unique targets preferentially expressed in mature and maturing cnidocytes, suggesting that lineage-specific gene expression is concentrated at terminal differentiation stages when the specialized structural components of the capsule and harpoon are assembled^16^. Comparative analysis across phylogenetically informative cnidarian species revealed that approximately 40% of *Nematostella* cnidocyte-expressed genes lack detectable homologs in other cnidarians under the similarity thresholds applied, while 95% of identified homologs are enriched in cnidocytes within their respective lineages^43,44,56^. Homologs were found for genes in all six regulatory cascades across all species examined, consistent with the presence of all six cascades in the stem cnidarian, though the pan-cnidarian conservation of cascade components regulated by *NvfoxE-like* raises the question of what transcription factors drive equivalent cascades in non-hexacorallian lineages. Collectively, these findings are consistent with a model in which cnidocytes arose in the stem cnidarian and diversified through lineage-specific expansion of the cnidocyte transcriptome, with newly recruited lineage-specific genes integrating with the conserved core program rather than replacing it to drive the morphological diversity of cnidocyte phenotypes observed across Cnidaria^11,16^.

## Discussion

*paxA* and *znf845* orthologs are broadly conserved in cnidarian genomes and regulate cnidocyte development in *Nematostella*^7,59^. *znf845* is a cnidarian innovation, and thus it has been hypothesized to likely play a central role in the emergence of cnidocyte fates early in cnidarian evolution. Exploiting the availability of scRNA-seq atlases from multiple species, we confirmed that *znf845* and *paxA* orthologs are exclusively expressed or highly enriched in cnidocytes and their progenitors across cnidarians, providing additional support that *znf845* and *paxA* are likely key players in the emergence of cnidocyte fates (Figure S3)^37,43,44,56^. This is further supported by the fact that *NvpaxA* positive targets largely overlap with the *Nvznf845* positive targets, and their roles in *Nematostella* appear to be to promote nematocyte development (Figure 3 H, J, K, and L). Because nematocytes are the only cnidocyte type universally present across all cnidarians^17,65^, this pattern of conservation is consistent with the *znf845*→*paxA* pathway representing a conserved core cnidocyte specification cascade across Cnidaria. Most of the *Nvznf845* targets are not shared with *NvpaxA*, which, combined with its role upstream of *NvpaxA*, further suggests its emergence was the critical, if not necessary, step towards the emergence of cnidocytes.

One hypothesis for the emergence of cnidocytes is that they evolved from a neuronal subtype, which was supported by initial findings that *Nvznf845* repressed neuronal markers in *Nematostella*^7^. However, our findings question this hypothesis. First, many of the “neuronal” genes that are repressed by *Nvznf845* have since been found to be expressed in both neurons and maturing cnidocytes^36^. Second, the bulk RNA-seq data we generated reveal that *Nvznf845* represses a greater number of genes in an uncharacterized immune cell population than in neurons (Figure S3J), and much less compared to neurons and gland cells. Thus, the model that cnidocytes emerged from neurons based on repression of neuronal fates by *znf845* is not supported by the current data. However, it should be noted that our findings do not refute the hypothesis that cnidocytes evolved from neurons but rather suggest it is premature to rule out alternative models for their emergence. The intriguing finding that *Nvznf845* represses a greater number of genes in an uncharacterized immune cell population than in neurons raises the possibility that cnidocytes may share a deeper evolutionary relationship with ancient secretory or immune cell types than currently appreciated a question that warrants further investigation.

Although all cnidarians share a common basic cnidocyte architecture of a capsule containing an inverted tubule that everts explosively upon stimulation, cnidocyte phenotypes have diversified considerably across the phylum. Some features map cleanly to the phylogeny, providing simple evolutionary hypotheses — for example, spirocytes are restricted to hexacorallians and ptychocytes to cerianthids, while at the ultrastructural level medusozoan nematocysts bear an operculum, non-actiniarian anthozoan nematocysts have an apical cap, and apical flaps are restricted to Actiniaria^11,16,17^. However, other aspects of cnidocyte phenotypic diversity do not map cleanly onto cnidarian phylogeny. Nematocytes, which are present in all cnidarians and are the most common cnidocyte in most species, vary widely in harpoon morphology, armature, function, and venom composition in a manner that lacks a clear phylogenetic signature, implying that much of this diversity arose independently within different lineages^11,16^. Importantly, cnidocyte subtypes as currently defined are based primarily on broad morphological criteria — capsule morphology, tubule armature, and harpoon structure — that incompletely capture this functional and biochemical diversity^11^, which likely explains the absence of a subtype-specific enrichment pattern in our atlas mapping analysis. This pattern of lineage-specific diversification is reflected in our gene conservation findings: based on reciprocal BLAST analysis across representative species including *Exaiptasia diaphana* (Figure S4), only ~25% (225/989 genes) of the genes we identified are conserved across cnidarians, ~50% (504/989 genes) are conserved within hexacorals, and ~60% (596/989 genes) are conserved across actiniarians, with 40% (393/989 genes) of *Nematostella* cnidocyte-expressed genes lacking detectable homologs outside *Nematostella* under the thresholds applied (Figure S4C–H). Together, these findings suggest that a large proportion of cnidocyte gene diversity emerges through later lineage-specific branching events and that lineage-restricted expansions of the cnidogenic regulatory program likely contributed to the morphological diversification of cnidocytes observed across Cnidaria. If true, this hypothesis suggests that: 1) understanding the emergence of cnidocytes likely hinges on investigating only a conserved subset of the *Nematostella* cnidogenic regulatory network, and 2) identifying convergent cnidocyte phenotypes that arose independently across lineages may provide opportunities to investigate the molecular mechanisms driving convergent evolution.

Our identification of *NvfoxE-like* provides an illustrative example of how lineage-specific cnidocyte regulatory programs may emerge. The *foxE-like* gene emerged as a result of gene duplication within the hexacorals (Figure 1C, Figure S1), and its expression was not detected in *Stylophora* cnidocytes^44^. We therefore propose that after duplication, *NvfoxE-like* was co-opted into the cnidocyte lineage in a subset of hexacorals or possibly only in *Nematostella*, where it regulates only ~15% of the cnidocyte-expressed genes identified here. Approximately 21% (31 genes) of *NvfoxE-like* positive targets are not shared with *Nvznf845* or *NvpaxA*, and 16 of the 31 genes are unique to *Nematostella* (Figure 4E). The pan-cnidarian conservation of cascade components regulated by *NvfoxE-like* in *Nematostella* raises the question of what transcription factors regulate equivalent cascades in non-hexacorallian lineages, consistent with the possibility that regulatory rewiring of conserved effector programs may be a recurring feature of cnidocyte diversification across the phylum. One consistent trend among all *NvfoxE-like* positive targets is that their expression is overrepresented in mature or maturing cnidocytes. Because conserved genes are underrepresented at these same stages, we propose that lineage-specific gene expression is concentrated at later stages of cnidocyte development, when the core conserved structural components of the capsule and harpoon are being assembled^16^. This is the developmental window during which we argue diverse cnidocyte morphologies emerge, particularly the harpoon morphologies that vary widely in function, structure, and armature across cnidarian lineages^11,16^. While further studies will be needed to elaborate and test the hypotheses we present here, these findings provide a molecular framework in which a conserved core cnidogenic program is repeatedly elaborated through lineage-specific regulatory innovation to generate the phenotypic diversity of cnidocytes observed across Cnidaria.

The fact that only 23% of the cnidocyte genes are shared widely across cnidarians suggests that homologous cell types share less than a quarter of their transcriptomes. Cnidocytes are unique in that they are an established synapomorphy that arose relatively “late” in animal evolution after the divergence of ctenophores, sponges, and placozoans. Thus, cell types that arose at the base of the metazoans may share even less gene expression, which may make efforts to resolve the homology of contentious cells, like neurons that may have evolved multiple times^66,67^, difficult by gene expression similarity alone. Future work on cnidocyte specification across cnidarians may provide clues about what features might be the most informative when trying to resolve the homology, or lack thereof, for similar metazoan cell types.

## Supplementary Material

1
