# Supplementary material for: Lineage-specific elaboration of a conserved cnidogenic program drives cnidocyte diversification and illuminates cell type evolution
Source: Res Sq. 2026 Jun 30:rs.3.rs-10009230. Preprint. [Version 1] doi: 10.21203/rs.3.rs-10009230/v1 (PMC13345560; doi:10.21203/rs.3.rs-10009230/v1)
Supplement: 1 [file NIHPPRS10009230V1-supplement-1.pdf]

**Supplementary figures**

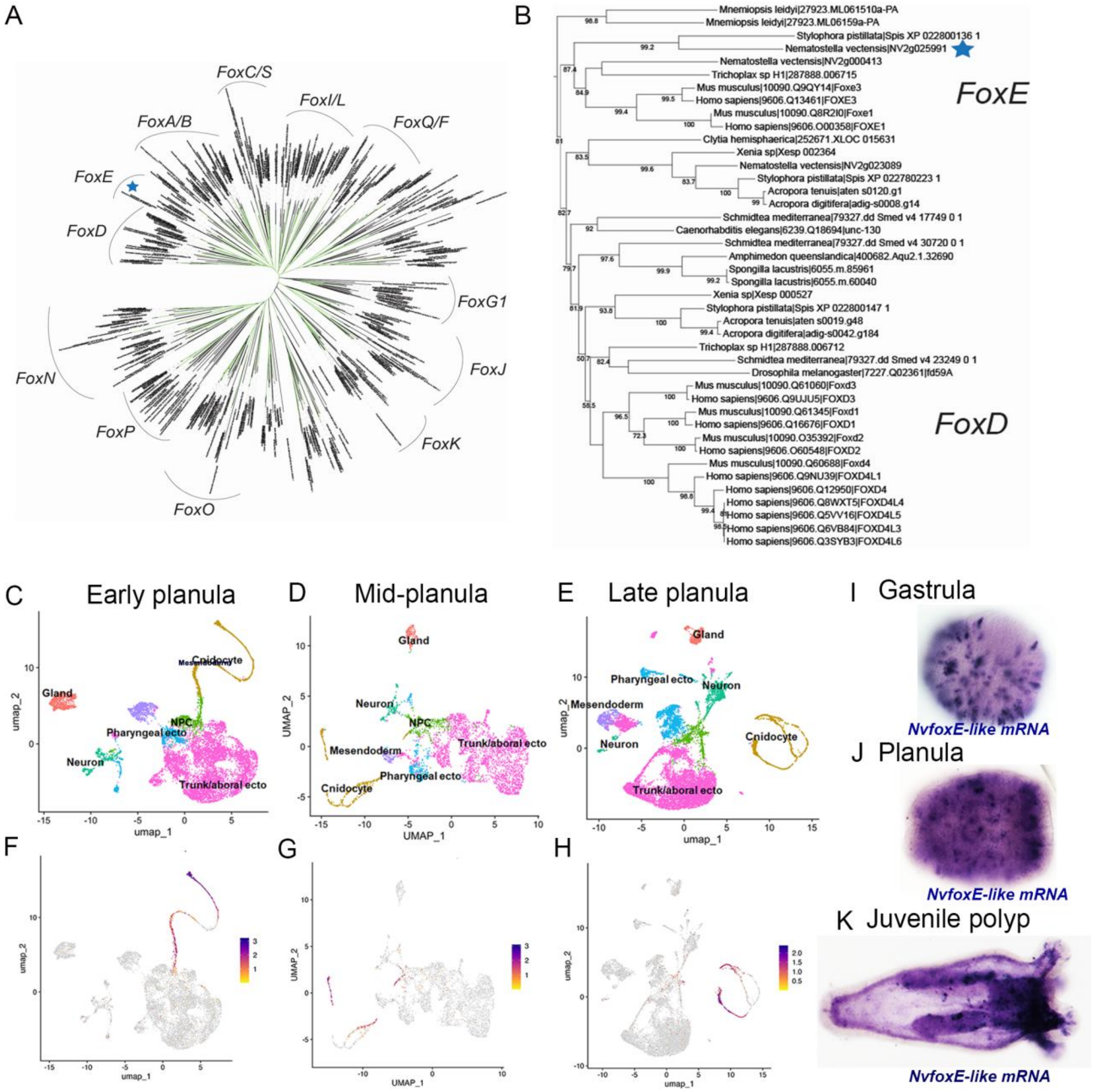


**Figure S1: *NvfoxD3-like* is more closely related to the FoxE family than FoxD and is expressed in cnidocyte lineages throughout development.** Phylogenetic gene tree of forkhead transcription factors across cnidarians and bilaterians, showing that *NvfoxD3-like* clusters with FoxE genes rather than FoxD (A). The blue star indicates the position of *NvfoxD3-like* within the tree. Zoomed-in view of the gene tree confirming that *NvfoxD3-like* is more closely related to the FoxE family than FoxD, supporting its renaming to *NvfoxE-like* (B). UMAPs showing annotated cell type clusters from scRNA-seq data at early planula (C), mid-planula (D), and late planula (E) stages. Feature-plots showing cell populations expressing *NvfoxE-like* at early planula (F), mid-planula (G), and late planula (H) stages. *In situ* hybridization showing the characteristic salt-and-pepper expression pattern of *NvfoxE-like* at gastrula (I), planula (J), and tentacle-bud (K) stages.

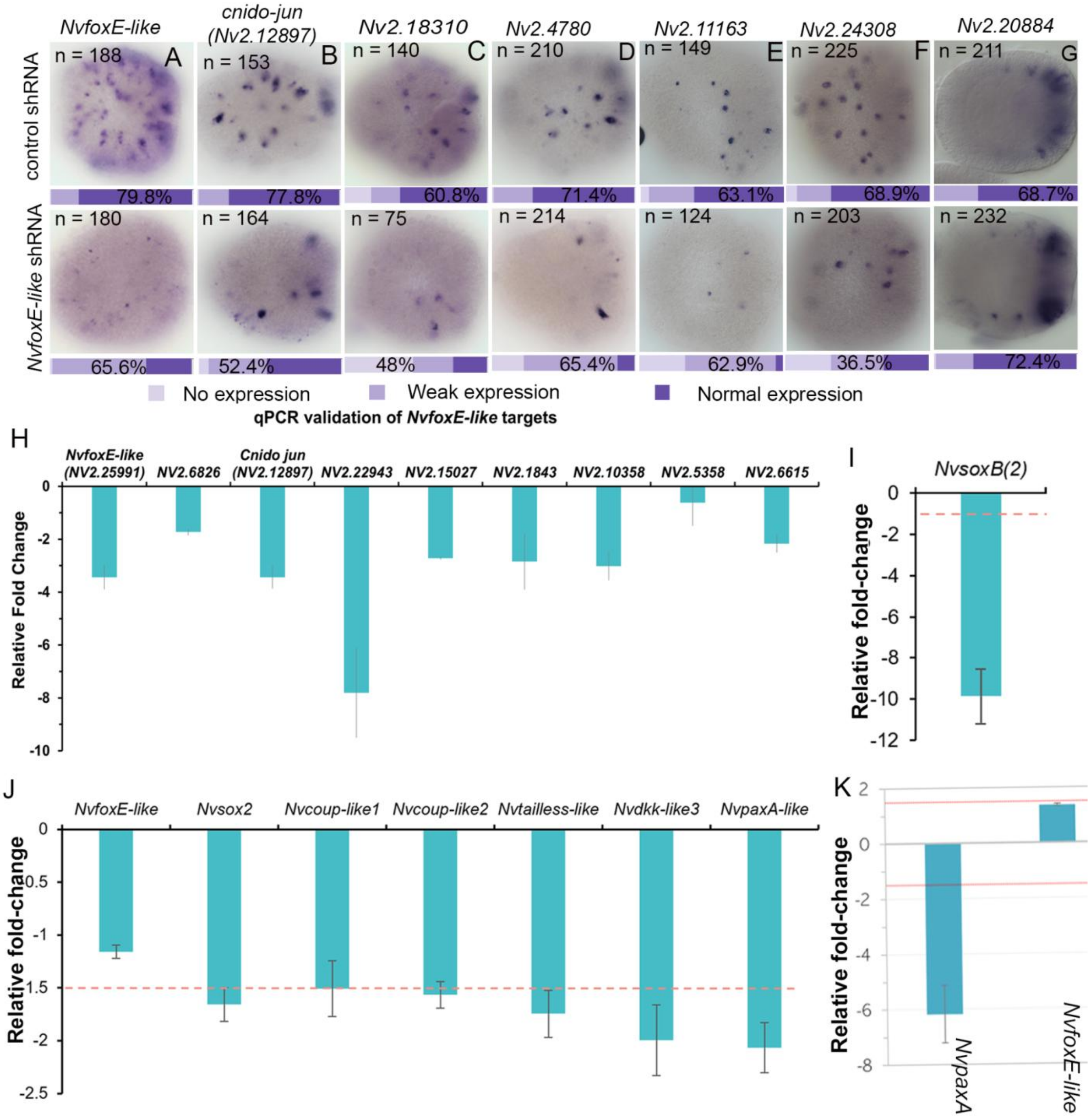


**Figure S2: Confirmation of *NvfoxE-like* targets identified by RNA-sequencing and additional data that *NvfoxE-like* is not downstream of *NvsoxB(2)*.** *In situ* hybridization (ISH) of identified targets confirm RNA-seq results (A–G). qPCR validation of *NvfoxE-like* targets confirms RNA-seq results (H). qPCR experiments were performed in triplicate using independent biological replicates. qPCR analysis of *NvsoxB(2)* knockdown in gastrula-stage embryos showing that *NvsoxB(2)* expression is significantly reduced in shRNA-injected animals, confirming effective knockdown (I). qPCR analysis showing that *NvfoxE-like* expression is not significantly affected by *NvsoxB(2)* knockdown, indicating that *NvfoxE-like* is not downstream of *NvsoxB(2)* in the cnidogenesis regulatory hierarchy (J). qPCR analysis showing that knockdown of *NvpaxA* does not significantly affect *NvfoxE-like* expression (K).

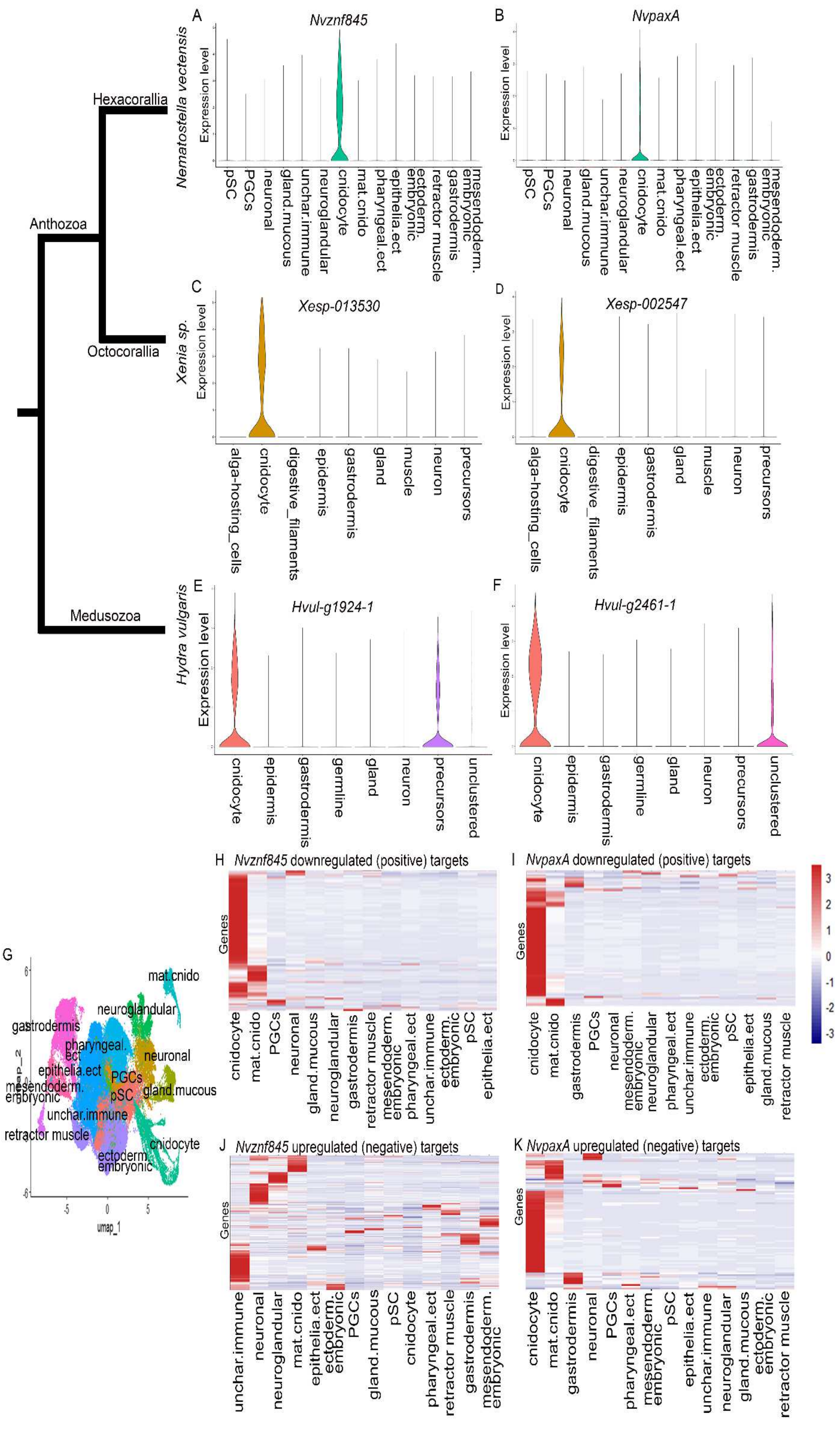

Hexacorallia
Anthozoa
Octocorallia
Medusozoa
Nematostella vectensis
Xenia sp.
Hydra vulgaris
A Nvznf845
B NvpaxA
C Xesp-013530
D Xesp-002547
E Hvul-g1924-1
F Hvul-g2461-1
Expression level
pSC
PGCs
neuronal
gland.mucous
unchar.immune
neuroglandular
cidocyte
mat.cnido
pharyngeal.ect
epithelia.ect
ectoderm. embryonic
retractor muscle
gastrodermis
mesendoderm. embryonic
alga-hosting_cells
cnidocyte
digestive_filaments
epidermis
gland
muscle
neuron
precursors
germline
unclustered
G
umap_1
umap_2
H Nvznf845 downregulated (positive) targets
I NvpaxA downregulated (positive) targets
J Nvznf845 upregulated (negative) targets
K NvpaxA upregulated (negative) targets
Genes

**Figure S3: *znf845* and *paxA* are conserved cnidocytes genes across Cnidaria.** Violin plots showing that single-cell expression of *Nvznf845* and *NvpaxA* are enriched in cnidocytes of the anthozoan *Nematostella vectensis* (A, B), and that their orthologs in the anthozoan *Xenia* sp. (C, D) and the medusozoan *Hydra vulgaris* (E, F) are similarly enriched in cnidocytes, suggesting conservation of cnidocyte-specific expression across cnidarian lineages. Single-cell sequencing atlas showing annotated cell types in *Nematostella vectensis* across embryonic and adult stages (G). Heatmaps showing that genes promoted by *Nvznf845* and *NvpaxA* are enriched mainly in cnidocytes in *Nematostella* (H, I). Genes repressed by *Nvznf845* are enriched in non-cnidocyte cell types, including uncharacterized immune cells, neurons, and neuroglandular cells (J), whereas genes repressed by *NvpaxA* retain enriched expression in cnidocytes and gastrodermis (K).

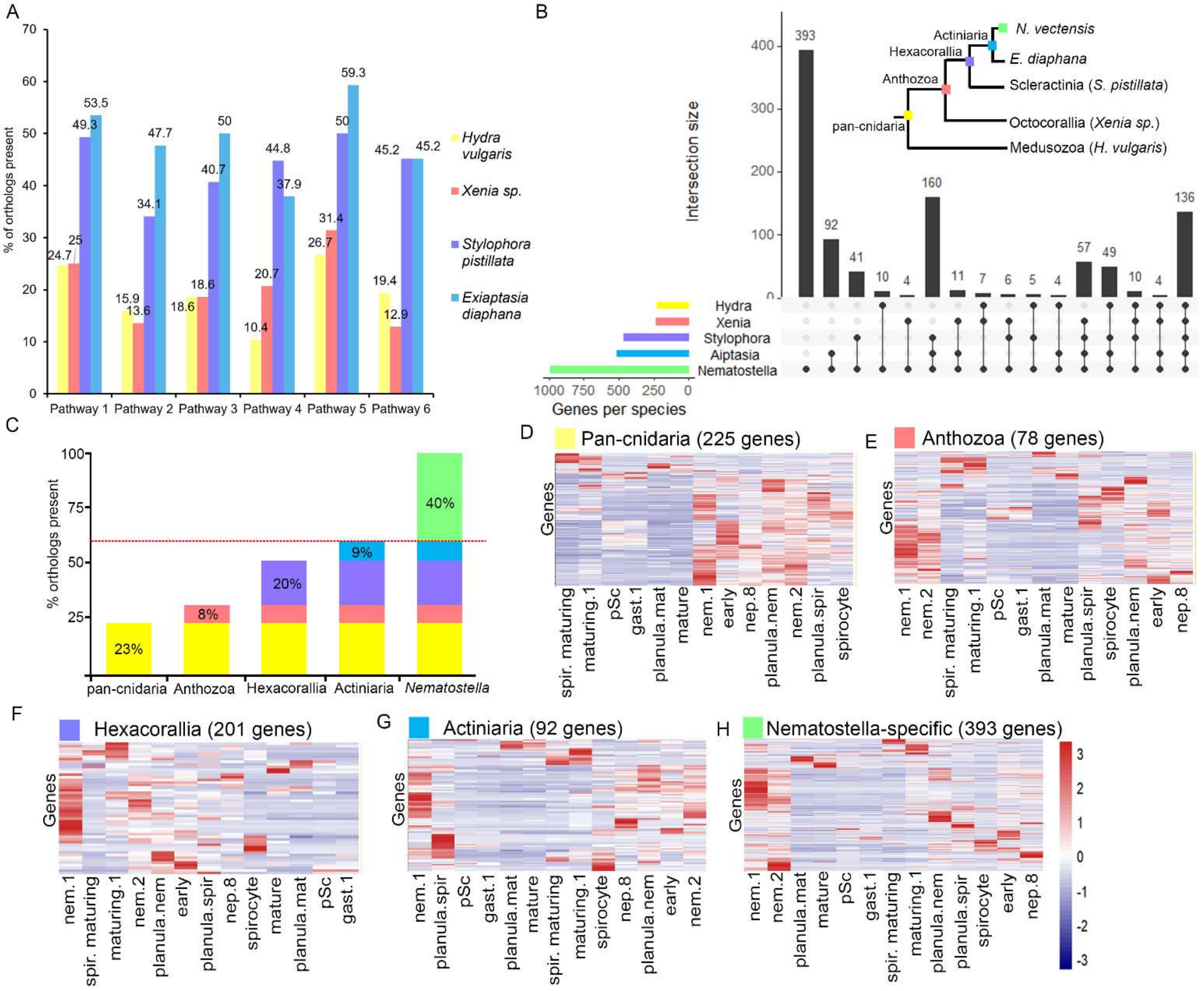


**Figure S4: Lineage-specific expansion of cnidocyte transcriptome contribute to cnidocyte diversity across cnidaria.** Bar graph showing the percentage of *Nematostella vectensis* cnidogenesis GRN orthologs for each of the six *Nematostella* pathways in *H. vulgaris*, *Xenia* sp., and *S. pistillata* (A). UpSet plot showing the overlap of identified orthologs among *Hydra vulgaris*, *Xenia* sp., *Stylophora pistillata*, and *Exaiptasia diaphana* (B). Schematic of cnidarian phylogeny illustrating the distribution of identified orthologs as pan-cnidarian, anthozoan-specific, hexacoral-specific, actiniaria-specific, or *N. vectensis*-specific (B- inset). Barplot showing percentage of genes added to cnidocyte transcriptome as cnidarians diverged (C). Heatmaps showing that both lineage-restricted and broadly conserved GRN genes are highly expressed across differentiated cnidocyte cell states, including nematocytes (Nep8+ cells, Nem1, Nem2, and planula nematocytes) and spirocytes (spirocytes, planula spirocytes, and maturing spirocytes), but show reduced expression in proliferating cells (pSC and gast.1) and matured cells (matured and planula matured) (D–H).

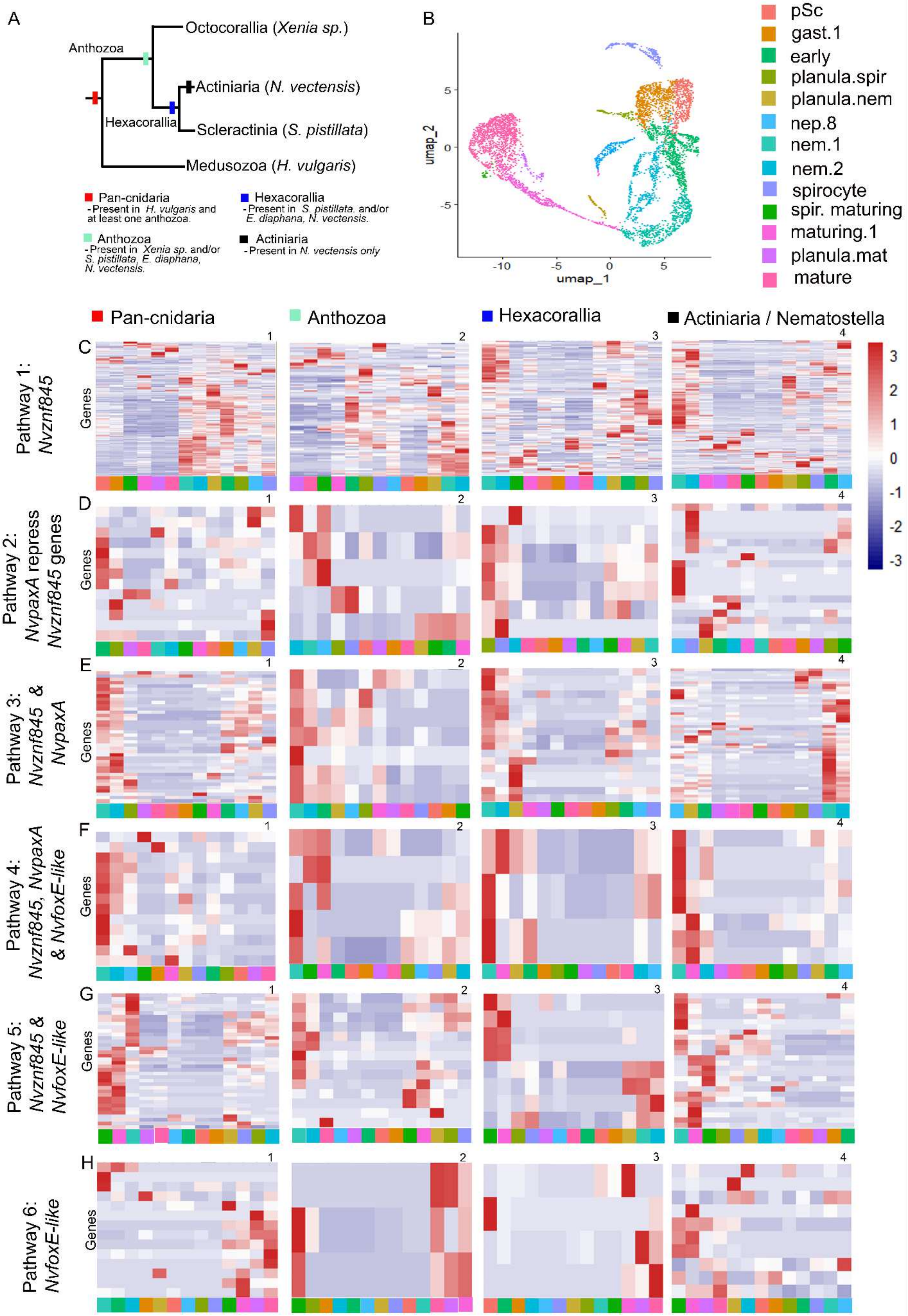

A
Octocorallia (Xenia sp.)
Anthozoa
Actiniaria (N. vectensis)
Hexacorallia
Scleractinia (S. pistillata)
Medusozoa (H. vulgaris)
Pan-cnidaria
-Present in H. vulgaris and at least one anthozoa.
Hexacorallia
-Present in S. pistillata. and/or E. diaphana, N. vectensis.
Anthozoa
-Present in Xenia sp. and/or S. pistillata, E. diaphana, N. vectensis.
Actiniaria
-Present in N. vectensis only
B
umap_2
umap_1
pSc
gast.1
early
planula.spir
planula.nem
nep.8
nem.1
nem.2
spirocyte
spir. maturing
maturing.1
planula.mat
mature
Pan-cnidaria
Anthozoa
Hexacorallia
Actiniaria / Nematostella
C
Pathway 1: Nvznf845
D
Pathway 2: NvpaxA repress Nvznf845 genes
E
Pathway 3: Nvznf845 & NvpaxA
F
Pathway 4: Nvznf845, NvpaxA & NvfoxE-like
G
Pathway 5: Nvznf845 & NvfoxE-like
H
Pathway 6: NvfoxE-like
Genes

**Figure S5: Co-option of lineage-specific genes into the conserved cnidogenic program drives lineage-specific cnidocyte phenotypes.** Schematic of cnidarian phylogeny illustrating the distribution of identified orthologs as pan-cnidarian, anthozoan-specific, hexacoral-specific, or actiniarian/*N. vectensis*-specific (A). Color legend showing cnidocyte cell types and cell states from scRNA-seq data (B). Heatmaps showing that orthologs of *N. vectensis* genes in the cnidogenic cascade promoted by *Nvznf845* are enriched in most cell types, including nematocytes and spirocytes, but show reduced expression in maturing and matured cnidocytes across Cnidaria (C1–C4). Pan-cnidarian orthologs of genes in the cascade promoted by *Nvznf845* but repressed by *NvpaxA* are enriched in spirocytes (D1), and this expression pattern is maintained (D2–D3) until the actiniarian lineage, where expression expands to include Nep8+ nematocytes in addition to spirocytes (D4). Orthologs of genes in the cascade co-regulated by *Nvznf845* and *NvpaxA* are enriched mainly in nematocytes (Nem1 and Nem2), and this expression pattern is conserved across Cnidaria (E1–E4). Orthologs of genes in the cascade co-regulated by *Nvznf845*, *NvpaxA*, and *NvfoxE-like* are enriched mainly in maturing cnidocytes (maturing.1 and spir. maturing) and Nem1 cnidocytes, and this expression pattern is conserved across Cnidaria (F1–F4). Orthologs of genes in the cascade co-regulated by *Nvznf845* and *NvfoxE-like* are enriched in maturing cnidocytes and Nem1 cells, and this expression pattern is conserved across Cnidaria (G1–G4). Orthologs of genes uniquely regulated by *NvfoxE-like* are enriched in maturing and matured cnidocytes, and this expression pattern is conserved across Cnidaria (H1–H4).

**Table S1: Distribution of published cnidocyte genes regulated by *NvfoxE-like*, *NvpaxA*, and N*vznf845*.** “+” indicates the corresponding transcription factor regulates the cnidocyte gene.

| Gene name | NV2 ID | NVE ID | *znf845* | *foxE-like* | *paxA* |
|---|---|---|---|---|---|
| ***znf845*** | *NV2.17877* | *NVE16303* | | | |
| ***sox2*** | *NV2.18482* | *NVE2102* | + | | |
| ***ncol1*** | *NV2.12621* | *NVE4226* | + | | |
| ***ncol3*** | *NV2.10686* | *NVE9976* | + | | |
| ***mcol1*** | *NV2.12623* | *NVE4225* | + | | |
| ***Ngal*** | *NV2.5200* | *NVE3845* | + | | |
| ***coup-like1*** | *NV2.23247* | *NVE21135* | + | | |
| ***coup-like2*** | *NV2.1477* | *NVE17305* | + | | |
| ***dkk3-like3*** | *NV2.10134* | *NVE13825* | + | | |
| ***NVE26200*** | *NV2.23901* | *NVE26200* | + | | |
| ***NVE5730*** | *NV2.21271* | *NVE5729* | + | | |
| ***NVE13546*** | *NV2.22446* | *NVE13546* | + | | |
| ***foxL2*** | *NV2.14459* | *NVE1324* | + | | |
| ***TX60B-like3*** | *NV2.11971* | *NVE21250* | + | | |
| ***ANTR2-like*** | *NV2.21578* | *NVE16011* | + | | |
| ***NEP3*** | *NV2.20490* | | + | | |
| ***myc4*** | | *NVE20964* | + | | |
| ***PRDM6-like*** | | *NVE25286* | + | | |
| ***cnido-jun*** | *NV2.12897* | *NVE16876* | + | + | |
| ***NVE15732*** | *NV2.24307* | *NVE15732* | + | + | |
| ***NVE15733*** | *NV2.24308* | *NVE15733* | + | + | |
| ***NVE3843*** | *NV2.5194* | *NVE3843* | + | + | |
| ***CALM3-like*** | *NV2.20419* | *NVE22513* | + | + | |
| ***GFI1B-like3*** | *NV2.25410* | *NVE16640* | + | + | |
| ***Ncol*** | *NV2.11820* | *NVE18955* | + | + | |
| ***foxE-like*** | *NV2.25991* | | | | |
| ***SoxA*** | *NV2.15027* | *NVE426* | | + | |
| ***cnido-fos*** | *NV2.19749* | *NVE5133* | | + | |
| ***ncol4*** | *NV2.11801* | *NVE18974* | + | | + |
| ***ggt*** | *NV2.1925* | *NVE20501* | + | | + |
| ***paxA*** | *NV2.6786* | *NVE25857* | + | | |
| ***NEP3-like*** | *NV2.20491* | *NVE22462* | + | + | + |
| ***NVE17236*** | *NV2.10392* | *NVE17236* | | | |
| ***ATF2-like1*** | *NV2.11500* | *NVE20585* | | | |
| ***myc5*** | *NV2.18928* | *NVE21040* | | | |
| ***Nkx2.2D*** | *NV2.11134* | *NVE10557* | | | |
| ***foxA*** | *NV2.11441* | *NVE20630* | | | |
| ***Six1-2*** | *NV2.11090* | *NVE9850* | | | |
| ***Tx60B-like5*** | *NV2.12610* | *NVE4235* | | | |
| ***NEP8*** | *NV2.19484* | *NVE15921* | | | |
| ***Max-like*** | | *NVE12845* | | | |
| ***PRDM13*** | | *NVE7535* | | | |

**Additional file 1:** List of primers

**Additional file 2**: Phylogenetic analysis of *NvfoxD3-like* across cnidaria and bilateria

**Additional file 3**: Phylogenetic tree for genes with the conserved forkhead box (fox) DNA binding domain across cnidaria.

**Additional file 4:** *Nvznf845, NvpaxA,* and *NvfoxE-like* RNA-seq differentially expressed genes DEGs

**Additional file 5:** List of genes in each cascade/pathway.

**Additional file 6:** Reciprocal best hit result of orthologs from *H. vulgaris, Xenia sp, S. pistillata,* and *E. diaphana*

**Additional file 7:** OrthoFinder result of orthologs from *H. vulgaris, Xenia sp,* and *S. pistillata*

# Supplementary Files

This is a list of supplementary files associated with this preprint. Click to download.

- Additionalfile2.pdf
- Additionalfile4.xlsx
- Additionalfile3.pdf
- Additionalfile6.xlsx
- Additionalfile1.xlsx
- Additionalfile5.xlsx
- Additionalfile7.xlsx
